# Coherent Phononics of van der Waals Layers on Nanogratings

**DOI:** 10.1021/acs.nanolett.2c01542

**Published:** 2022-08-12

**Authors:** Wenjing Yan, Andrey V. Akimov, María Barra-Burillo, Manfred Bayer, Jonathan Bradford, Vitalyi E. Gusev, Luis E. Hueso, Anthony Kent, Serhii Kukhtaruk, Achim Nadzeyka, Amalia Patanè, Andrew W. Rushforth, Alexey V. Scherbakov, Dmytro D. Yaremkevich, Tetiana L. Linnik

**Affiliations:** † School of Physics and Astronomy, University of Nottingham, Nottingham NG7 2RD, United Kingdom; ‡ CIC nanoGUNE BRTA, Tolosa Hiribidea, 76, 20018 Donostia-San Sebastián, Basque Country, Spain; § Experimentelle Physik 2, Technische Universität Dortmund, Otto-Hahn-Strasse 4a, 44227 Dortmund, Germany; ∥ Laboratoire d’Acoustique de l’Uiversité du Mans (LAUM), UMR 6613, Institut d’Acoustique - Graduate School (IA-GS), CNRS, Le Mans Université, 72085 Le Mans, France; ⊥ IKERBASQUE, Basque Foundation for Science, 48013 Bilbao, Basque Country Spain; # Department of Theoretical Physics, V.E. Lashkaryov Institute of Semiconductor Physics, Pr. Nauky 41, 03028 Kyiv, Ukraine; ∇ Raith GmbH, Konrad-Adenauer-Allee 8, 44263 Dortmund, Germany

**Keywords:** coherent phonons, van der Waals nanolayers, picosecond ultrasonics, phonon modes hybridization, hybrid nanostructures, pump−probe spectroscopy

## Abstract

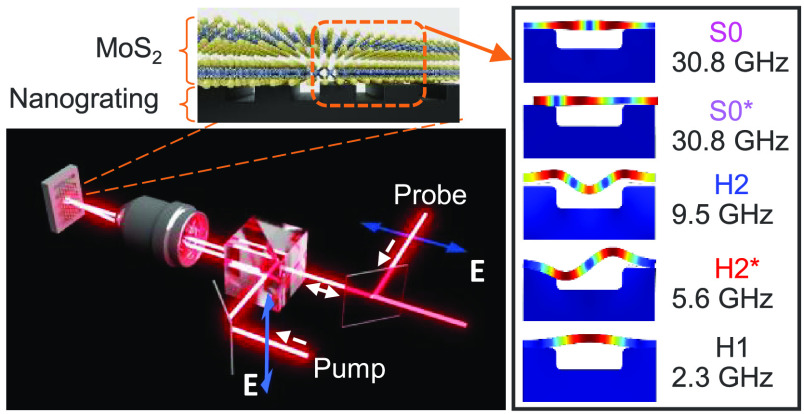

Strain engineering can be used to control the physical properties of two-dimensional van der Waals (2D-vdW) crystals. Coherent phonons, which carry dynamical strain, could push strain engineering to control classical and quantum phenomena in the unexplored picosecond temporal and nanometer spatial regimes. This intriguing approach requires the use of coherent GHz and sub-THz 2D phonons. Here, we report on nanostructures that combine nanometer thick vdW layers and nanogratings. Using an ultrafast pump–probe technique, we generate and detect in-plane coherent phonons with frequency up to 40 GHz and hybrid flexural phonons with frequency up to 10 GHz. The latter arises from the periodic modulation of the elastic coupling of the vdW layer at the grooves and ridges of the nanograting. This creates a new type of a tailorable 2D periodic phononic nanoobject, a flexural phononic crystal, offering exciting prospects for the ultrafast manipulation of states in 2D materials in emerging quantum technologies.

Two-dimensional van der Waals crystals (2D-vdW) consist of chemically bonded atomic layers held together by weak vdW forces. Thus, they provide the perfect atomic-size “Lego-type” toy models for the exploration of new classical and quantum phenomena in solid state physics.^1^ Their versatile homo- and heterostructures created by stacking, twisting, stretching and bending of vdW layers provide means of manipulating optical, electrical, magnetic, piezoelectric and spin properties. This has allowed the observation of a range of phenomena related to strain, such as strain-controlled band gap,^2,3^ luminescence^4−6^ magnetization,^7^ and single photon emission.^8−10^ These experiments form a prerequisite for the development of methods that employ coherent high-frequency phonons to control quantum excitations in vdW nanolayers on picosecond temporal and nanometer length scales. One of the main challenges for using phonon technology for the manipulation of the diverse physical parameters in 2D-vdW devices is to extend the operating phonon frequency from a few gigahertz (GHz) to higher values and correspondingly shorter wavelengths from micrometers to nanometers. However, coherent high frequency in-plane phonons with nanometer wavelengths have not yet been explored. Fundamental studies of such phonons in 2D-vdW layers are required to fully understand and exploit their interactions with single quanta of electrons, excitons, spins, magnons, and other excitations.

Here, we demonstrate a new hybrid nanostructure consisting of a vdW material and nanoscale gratings, as schematically shown in the inset of Figure 1a. By bringing the vdW layer into elastic contact with the nanograting we are able to generate and detect propagating coherent phonon modes with high frequency up to 40 GHz. Also, high-frequency immobile hybrid flexural phonon modes are generated due to coupling of phonons above the grooves and ridges of the nanograting. This new type of 2D periodic phononic nanoobject, a flexural phononic crystal, opens up exciting directions for exploitation of phonon hybridization effects in vdW materials at frequencies more than 1 order of magnitude higher than recently studied vdW based phononic crystals.^11,12^

**Figure 1:**
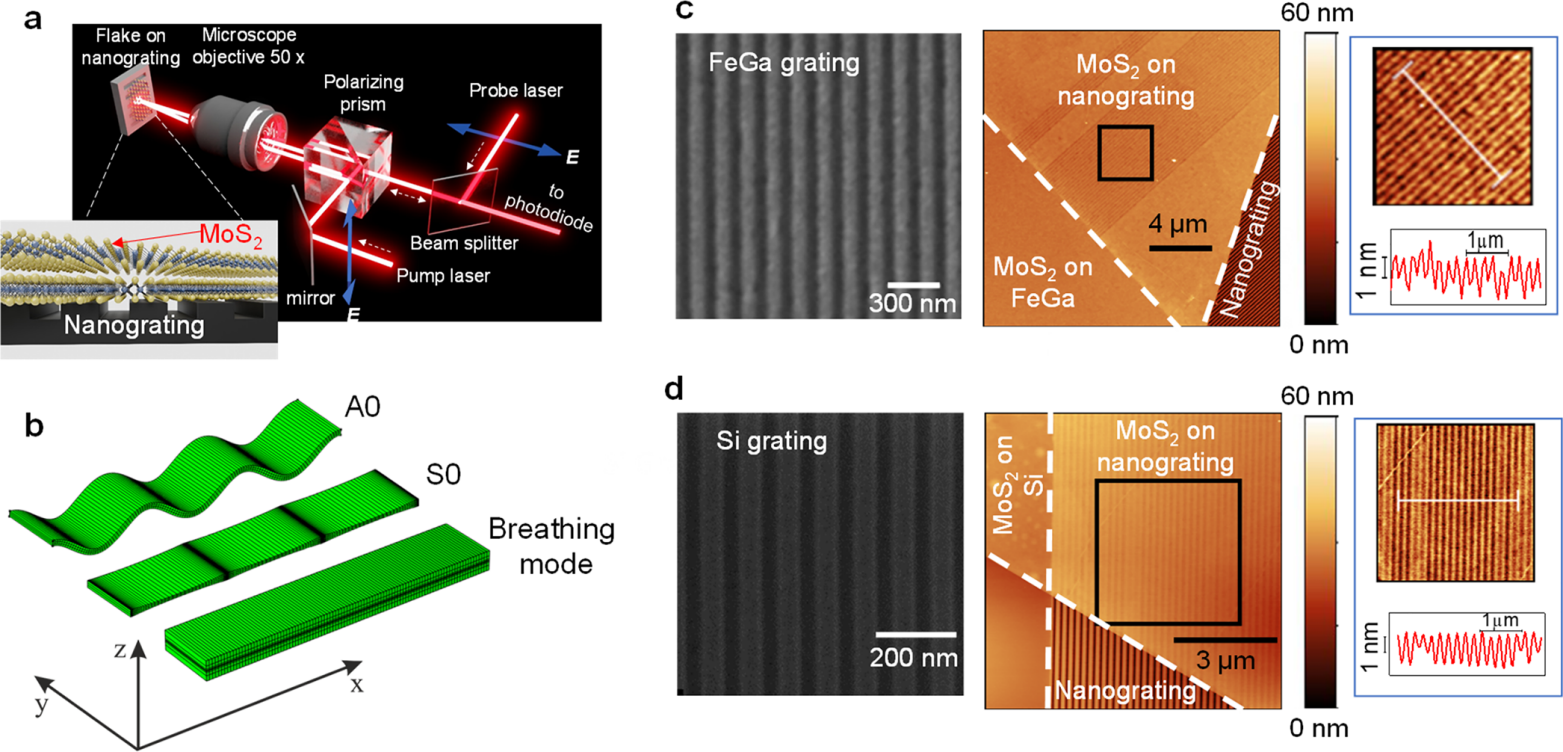
Picosecond acoustics of van der Waals layers on nanogratings. (a) Schematic illustration of the pump–probe setup and the layer on the grating. The blue arrows show the polarization of the light. (b) The simulation shows the fundamental antisymmetric (A0, top), symmetric (S0, middle) and breathing modes (bottom) of the free-standing layer. (c) Scanning electron microscopy (SEM) image of the FeGa grating with 150 nm period (left), AFM image of a 200 nm period nanograting with a 13 nm thick MoS_2_ (middle). A zoomed image of the area enclosed by the black square is shown to the right, and the height profile of the layer on the grating along a white line is shown below. (d) The same as in panel c but for the Si grating: SEM (period 100 nm); AFM (layer thickness 8.3 nm on grating with period 200 nm).

As discussed below, our research goes beyond the investigation of phonon modes in free-standing vdW layers,^13^ which are well-known to consist of a number of symmetric (S) and antisymmetric (A) elastic Lamb waves with frequency ω(**q**), where **q** is the in-plane wavevector.^14^ The two upper images in Figure 1b illustrate the displacement of atoms in the lowest antisymmetric (A0) and symmetric (S0) Lamb modes. For small wavevectors *q*_*x*_ ≪ 2π/*a* (*a* is a thickness of the layer), the A0 and S0 modes possess quadratic and linear dispersion given by *ω ∼ q*_*x*_^2^ (for A0) and ω = *s*_||_*q*_*x*_ (for S0), respectively, where *s*_||_ is an in-plane sound velocity.^14^ These are often referred to as flexural and longitudinal (LA) phonon modes, respectively. The lower panel in Figure 1b also shows the symmetric breathing mode. This phonon mode has frequency ω = *πs*_⊥_/*a*, where *s*_⊥_ is the sound velocity in the direction perpendicular to the layer and is localized in the layer as in an acoustic cavity.^15,16^ Phonons in free-standing vdW layers have been studied in a number of recent experiments using incoherent (spontaneous Brillouin scattering) and coherent phonon techniques.^15−17^

For our studies, we used the vdW crystal MoS_2_^18^ and various nanogratings, fabricated by focused ion and electron beam lithography, on Si substrate, Cr and SiO_2_ films, and a ferromagnetic 100 nm-thick FeGa film on a GaAs substrate.^19^ The MoS_2_ flakes with thicknesses from 3 to 30 nm were transferred onto the nanogratings using a viscoelastic transfer technique^20^ (Supporting Information, Section 1) and characterized by atomic force microscopy (AFM). Figure 1c,d shows examples of the scanning electron microscope (SEM) images for bare FeGa and Si gratings, respectively, with periods *d*_FeGa_ = 150 nm and *d*_Si_ = 100 nm. AFM images of the hybrid structure formed by the nanograting and vdW flake are also shown in Figure 1c,d. The AFM zoomed regions demonstrate that flakes with thicknesses of ∼10 nm form a conformal coating of the nanograting with a height modulation of ∼1 nm. Such modulation is smaller than the depth of the grooves (>10 nm) and indicates a good mechanical contact due to attractive electrostatic forces between the MoS_2_ flake and the nanograting.

The schematic of the pump–probe experiments for generation and detection of coherent 2D phonons in the hybrid vdW layer/grating is shown in Figure 1a (Supporting Information Section 2). The generation of phonon by the pump pulse in this geometry is governed by the stress induced by the photoexcited carriers in MoS_2_^21^ and thermal stresses generated in the nanogratings upon the absorption of light. The detection of generated phonons by the probe pulse is governed by the photoelastic effect.^22,23^ For a grating of period *d* along the *x*-axis, the spectrum of the measured phonons in *q*_*x*_-space is concentrated near *q*_*x*_*= nG*, where *G* = 2π/*d* is the projection of the reciprocal grating vector **G** along the *x*-axis and *n* is an integer, all folded in accordance with the Bloch-Floquet theorem to the center of the Brillouin zone of the periodic nanostructure. The measurements are performed with the probe laser polarization **E** parallel to the vector **G** (**E** ∥ **G**) or perpendicular to **G** (**E** ⊥ **G**). Pump–probe signals, measured in the temporal domain with subpicosecond resolution, show oscillations due to coherent phonons generated by the pump pulse. The spectral analysis of the measured signals in various temporal intervals and comparison of the experimental phonon spectra with the theoretical models for the phonon dispersion allow us to identify the nature of the detected phonon modes.

Figure 2 illustrates the method for obtaining the phonon spectrum from the measured temporal dependence of the reflectivity signal Δ*R*(*t*). As an example, we show the results for two MoS_2_ layers on FeGa gratings. The left insets in Figure 2 show the pump–probe raw data signals measured in the temporal interval of ∼1 ns. The signals show strong slow oscillations with frequency *f*_1_and weaker faster oscillations. To analyze the spectral features in detail, we subtract the slow oscillating background and consider the signals in the time interval of 500 ps after the pump pulse and their fast Fourier transforms (FFT) shown in the main panels of Figure 2 and in the right insets, respectively. The phonon induced signals depend on the electric field polarization **E** of the probe pulse relative to the orientation of the grating. The spectral lines with *f*_G_ = 12.6 GHz and *f*_G_ = 16.5 GHz in the samples with *d* = 200 and 150 nm, respectively, have the highest amplitude and are also detected in the bare gratings (i.e., without MoS_2_ layer). These frequencies correspond to the Rayleigh-like waves in the FeGa grating.^19^ Other spectral lines, with frequencies *f*_2_ (detected when **E⊥G**) and *f*_3_ (detected when **E||G**), have lower amplitude, but were observed only when probing the MoS_2_ nanolayer on the grating. Thus, we conclude that the phonon modes with frequencies *f*_1_, *f*_2_, and *f*_3_ correspond to the phonons of the hybrid nanostructure. For MoS_2_ layers transferred on gratings based on Si and Cr, the signals are similar but the three modes are not always detected. In the short period Si (*d* = 100 nm) and Cr (*d* = 50 and 100 nm) gratings, the signals show only the mode with the lowest frequency *f*_1_. The layers transferred on SiO_2_ gratings did not reveal any oscillation due to coherent phonons except the high-frequency breathing mode described elsewhere.^15,16^

**Figure 2:**
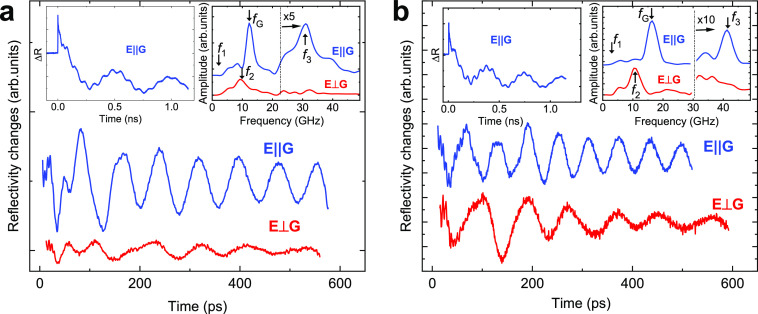
Pump–probe signals. Temporal evolutions of the measured reflectivity pump–probe signals *ΔR*(*t*) after the subtraction of the slow oscillating background in two hybrid nanostructures: (a) 8 nm thick MoS_2_ layer transferred on 200 nm period FeGa nanograting; (b) 10 nm thick MoS_2_ on 150 nm-period FeGa nanograting. The left insets show the raw measured signals including the slow oscillating background, and right insets are the fast Fourier transforms of *ΔR*(*t*) presented at the main panels. The vertical arrows in the right insets indicate the frequencies of phonon modes measured in the experiments.

Figure 3a shows the measured frequencies as a function of ***G*** = 2π/*d*. Here, the frequencies are classified into three groups: *f*_1_, closed circles; *f*_2_, crosses; and *f*_3_, open circles. The vertical size of the symbols characterizes the frequency uncertainty defined by the temporal window in the FFT. The solid black straight line is the theoretical dispersion of the S0 mode in a free-standing MoS_2_ layer with *s*_||_ = 6 km/s (for elastic parameters, see Supporting Information, Section 4). It can be seen that the experimental values of *f*_3_ fit well the S0 mode for free-standing MoS_2_. Thus, we assign coherent oscillations with frequency *f*_3_ to the symmetric S0 phonons. To support this statement, we show in the inset of Figure 3a the temporal signal after high pass filtering. Oscillations with a period of 24 ps are clearly seen, which correspond to the frequency *f*_3_ = 42 GHz which agrees with the frequency of the S0 mode. For earlier times (*t* < 100 ps, see inset of Figure 3a), lower amplitude oscillations with a period ∼5 ps appear on the background of the slower oscillations, which results in FFT peak at 170 GHz. We attribute these high-frequency oscillations to the breathing mode with *f* = *s*/2*a* (*s* ∼ 3200 m/s, out of plane longitudinal sound velocity) reported in previous works.^16^ The identification of the S0 mode and its agreement with the mode for a free-standing vdW layer is the first important conclusion of the present work.

**Figure 3:**
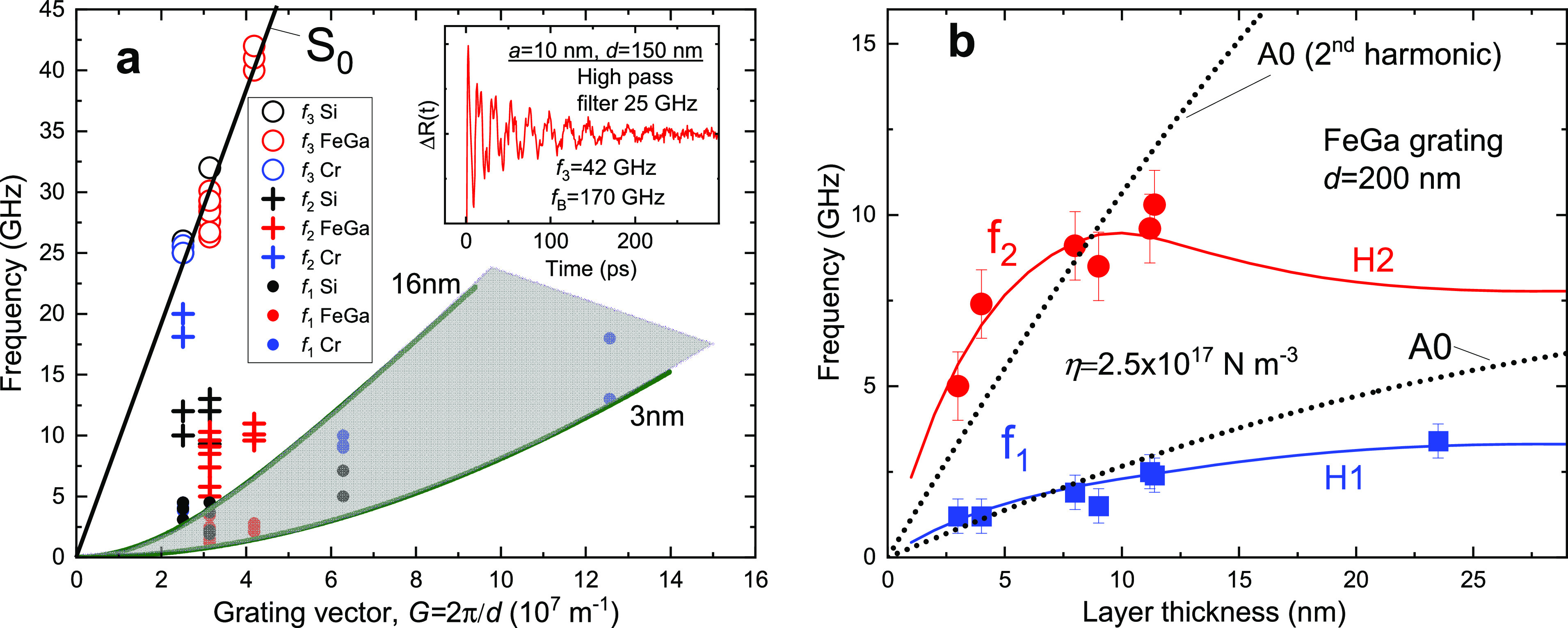
Phonon modes in hybrid nanostructures. (a) The measured frequencies of phonon modes as a function of grating vector for various hybrid nanostructures (symbols). The correspondence of different symbols to the frequency group (*f*_1_, *f*_2_ and *f*_3_) and grating material (FeGa, Si, and Cr) are shown in the inset table. The solid lines are the calculated dispersion curves for S0 (black straight line) for the layers with a <20 nm and A0 modes (green curved lines) for the free-standing MoS_2_ layers. The shaded area includes phonon modes calculated for A0 modes in the layers with the thicknesses between 3 and 16 nm. The inset shows the temporal evolution of the measured *ΔR*(*t*) after high pass filtering which emphasizes the detection of high frequency S0 and breathing modes. (b) The dependence of frequencies *f*_1_ and *f*_2_ on the vdW layer thickness measured in FeGa grating with a period *d* = 200 nm (symbols) and the corresponding theoretical dependences H1 and H2 (lines) calculated by Comsol Multiphysics software. The dotted lines are the calculated thickness dependences for A0 modes with *q*_*x*_ = *G* and *q*_*x*_ = 2*G* in a free-standing layer.

We now discuss the lower frequency range 1–11 GHz corresponding to *f*_1_ and *f*_2_. The shaded area between the two curved green solid lines in Figure 3a corresponds to the frequency range for A0 modes for free-standing MoS_2_ layers with various thicknesses from 3 to 16 nm. The measured values of *f*_1_ (filled circles) fit the shaded area; also, the measured values of *f*_2_ (crosses) fit the frequency area for the second harmonic (*n* = 2) for A0. However, the dependences of *f*_1_ and *f*_2_ on the layer thickness does not agree with the theoretical predictions for free-standing layers. This disagreement is clearly seen in Figure 3b, which compares the measured dependences *f*_1,2_ (*a*) for the 200 nm period FeGa gratings (symbols) with the theory (dotted lines). Thus, we conclude that phonon modes with *f*_1_ and *f*_2_ cannot be due to modes in a free-standing vdW layer and that further analysis should treat the MoS_2_/grating as a whole to take into account the elastic contact between the layer and the ridges of the grating.

To describe the elastic contact at the MoS_2_/grating interface, we model the interface as an ensemble of springs with stiffness per unit area, η (see the inset in Figure 4a). To estimate the value of η in our hybrid nanostructure, we perform experiments in back geometry when the pump pulses excite the nanograting from the back of the substrate (Supporting Information, Section 1). In this geometry, the vdW layer is excited only by coherent phonons transmitted through the nanograting to the layer. From the analysis of these experiments, we estimate η ∼ 10^17^–10^18^ N m^–3^, which is consistent with earlier studies of vdW layers on planar substrates.^24,25^

**Figure 4:**
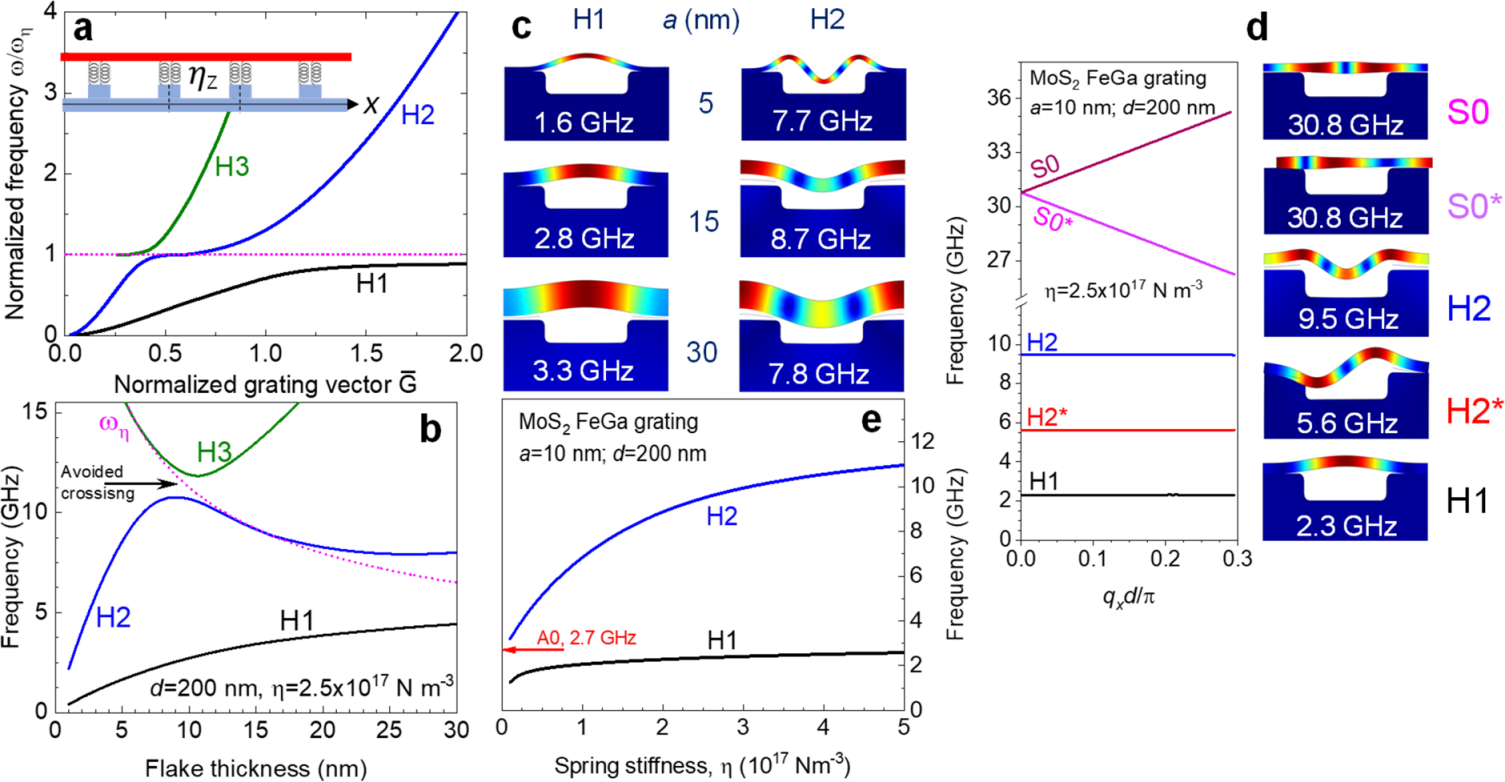
Theory of phonon mode hybridization. (a) The dependence of normalized frequencies of the experimentally relevant hybrid eigen modes on the normalized grating vector *G̅* in the hybrid nanostructure. For small *G̅* the H1 and H2 modes are the flexural wave resonances above the grooves, while H3 is the mode above the ridges dominated by mass-on-springoscillation whose frequency is shown by dashed dotted line. With increasing *G̅*, hybridization of these modes takes place. For large *G̅* all these modes are distributed over both the grooves and the ridges (H1 is dominated by the laterally unmodulated mass-on-spring oscillation, while H2 and H3 by flexural waves). Inset: the scheme of the spring model. (b) Calculated dependence of the mode frequencies on the layer thickness. The avoided crossing of modes H2 and H3 is highlighted by an arrow. The dotted line corresponds to the mass-on-spring type oscillation. (c) The layer motion for H1 and H2 hybrid modes displayed for three layer thicknesses. It shows how modes localized above the groove start to penetrate onto the ridges of grating with the increase of *a*. (d) The dispersion curves and layer motion for the phononic crystal showing even (S0, H1, and H2) and odd (S0* and H2*) phonon modes near the center of the Brillouin zone. **(**e) The dependences of the frequencies for H1 and H2 modes on the spring stiffness. The arrow indicates the frequency of A0 resonance for free-standing MoS_2_ layer. Panels a and b are the result of analytical calculations for infinitively rigid grating and *q*_*x*_*a* ≪ 1; other panels are numerical calculations performed by COMSOL Multiphysics for FeGa grating with *d* = 200 nm.

For a qualitative analysis of phonon modes in our hybrid nanostructure, we have developed an analytical model (Supporting Information, Section 3). We assume *q*_*x*_*a* ≤ 1 and treat our periodic nanostructure as a 2D phononic crystal for flexural waves propagating laterally above the infinitively rigid grating with the same width of grooves and ridges as in the experiment. The acoustic fields in the layer are determined by the wave equations, different over the grooves and the ridges, which are coupled by the boundary conditions. As a result this leads to the eigen modes of two groups of infinitive number of phonon branches: the low frequency (ω < ω_*η*_) group, which starts at ω = 0 and *G* = 0, and the high-frequency (ω > *ω*_*η*_) group, where elastic modes start at ω = *ω*_*η*_. Here,  is the frequency of the laterally unmodulated mass-on-spring oscillations of the layer connected to the ridge by the springs and ρ is the density of the vdW layer. Figure 4a shows the calculated normalized eigen frequencies Ω ≡ ω/*ω*_*η*_ for the three lowest even (relatively to the center of the groove) modes H1, H2, and H3 from these groups as a function of the normalized grating vector *G̅* ∼ *G*. More phonon modes are shown in Figure S2. For *G̅* ≪ 1, these modes in the low- and high-frequency branches are predominantly localized on the grooves and ridges, respectively. With decreasing period of the structure, *G* and ω increase and the highest modes in the lower frequency branch start to interact with the lowest modes in the high frequency branch. This results in the repulsion (hybridization) of these modes clearly seen in Figure 4a between the modes H2 and H3 and, at higher *G*, between the lowest branch H1 and H2. The mode repulsion and corresponding avoided crossing gap is clearly seen also in the dependence of the frequency on the layer thickness *a*, as shown in Figure 4b for specific grating parameters.

In short period gratings (*G̅* → ∞) the influence of elastic contact (springs) on the flexure-dominated modes becomes negligible and the two upper modes H2 and H3 become identical to the flexural waves with *q*_*x*_*= G* and *q*_*x*_*=* 2*G* in the free-standing layer, while the lowest mode H1 becomes dominated by laterally homogeneous mass-on-spring motion with Ω_H1_ saturating at about Ω = 0.9. The asymptotics and analytical equations for Ω(*G̅*) at *G̅* → ∞ and *G̅* → 0 are presented in the Supporting Information, Section 3.

The main conclusion of this theoretical analysis is that for 0.1 ≤ *G̅* ≤ 1, which corresponds to our experiments with η ∼ 10^17^–10^18^ N m^–3^, the hybridization of phonons above the grooves and ridges essentially leads to the significant deviation of the phonon eigen frequencies of the complete structure from those in the free-standing layers or plain layers on springs.^26^

To model the actual hybrid nanostructures, as used in the experiments, we use the COMSOL Multiphysics software. Figure 4c shows examples of the numerically calculated layer motion for the two lowest phonon modes H1 and H2 for different flake thicknesses *a* on the FeGa grating. It illustrates how the modes vary and the hybridization takes place with the increase of flake thickness. Thus, there is a qualitative agreement with the analytical predictions that the flexure-dominated modes, which are localized above the grooves for thin layers, extend to the ridges and incorporate the mass-on-spring motion over the complete structure at larger *a*.

The calculated dispersion curves near the center of the Brillouin zone and the corresponding motion of the layer are shown in Figure 4d for the MoS_2_ layer with *a* = 10 nm on the FeGa nanograting and η = 2.5 × 10^17^ N m^–3^. Here, we show even and odd phonon modes. More phonon modes are discussed in Supporting Information, Section 4. In pump–probe experiments, odd modes marked with the star are much less efficiently excited and detected than even modes because the width of the pump laser beam significantly exceeds the period of the grating. It can be seen that for the used parameters, the lowest three modes with frequency H1, H2, and H2* have a flat dispersion and hence have extremely small group velocities. In contrast, the zeroth-order Lamb modes S0 and S0* are fast propagating modes and their frequency in the zone center is almost the same as in the free-standing layer. It is worth mentioning that in agreement with the theory of phononic crystals, there is a phonon stop band between the even and odd modes H2 and H2*, respectively.

To compare the numerical analysis with the experiment we use the only fitting parameter, the spring stiffness η. Figure 4e shows the dependence of the H1 and H2 frequencies for the 10 nm MoS_2_ layer on a FeGa grating with *d* = 200 nm. In the range of η from zero up to 10^18^ N m^–3^, the phonon frequencies *f*_1,2_ cover the range up to 12 GHz. Thus, each of the experimentally measured frequencies pair *f*_1_ and *f*_2_ displayed in Figure 3a can be compared with the calculated H1 and H2 pair for a given fitting value of η, which ranges from 1.6 × 10^17^ to 7.6 × 10^17^ N m^–3^. It is interesting that the value of η does not vary strongly with the flake thickness for the nanolayers on the FeGa grating. This is clearly demonstrated in Figure 3b where the measured (symbols) and calculated (solid lines) dependencies of the frequency on layer thickness, *f*_1,2_ (*a*) and *f*_H1,H2_ (*a*), respectively, for the FeGa grating are compared. There is a good agreement between experimental data and theoretical curves for a fitting parameter η = 2.5 × 10^17^ N m^–3^, which confirms a phonon hybridization effect for flexural modes. The earlier mentioned fact that not all samples show both hybrid modes may be explained by the value of η which results in the phonons with frequencies *f*_1_ and *f*_2_ far from the hybridization regime. Then only the mode which has the highest amplitude is detected. The uniform value of η for the nanolayers on FeGa gratings suggests a homogeneous adhesion at the layer/substrate interface due to specific electrostatic properties generated during the FIB nanofabrication.

In conclusion, we have fabricated hybrid nanostructures which consist of vdW nanolayers transferred onto gratings with periods from 50 up to 250 nm. We have revealed three modes in the GHz frequency range for the coherent phonons: (i) a thickness independent zeroth-order symmetric S0 Lamb mode with frequency up to ∼40 GHz over the grating with a period of 150 nm; (ii) two hybrid flexural phonon modes, which combine flexural modes above the grooves and modes with the mass-on-spring oscillations above the ridges of the nanograting. For the considered values of elastic coupling with the ridges, η ≪ 10^18^ Nm^–3^, the hybrid modes of the layers are standing while S0 phonons propagate with the same velocity as in the free-standing layers. These findings pave a way to control strain induced processes in 2D-vdW materials on a picosecond time scale. It has been shown that the amplitude of a compressive strain pulse in picosecond ultrasonic experiments can reach several percent^27−30^ and so coherent phonons injected into a hybrid nanostructure, such as those described in this paper, may cause displacements on the nanometre scale. Such high excitation, when confined in the 2D-vdW layer can trap carriers and change locally the transport and optical properties on a picosecond time scale. Based on static and low frequency strain-induced effects, high frequency phonons could be used in a similar way as done in epitaxial nanostructures to manipulate light emission (for review see^31^), conductivity,^32−34^ magnetization^35,36^ and plasmons^37^ on a picosecond time scale. The propagating S0 phonons could play a role in the realization of a quantum “bus” idea where phonons are used to transport information between qubits.^38^ The ability of phonons to manipulate and communicate quantum excitations will undoubtedly make such devices ideal candidates for hybrid quantum devices and for quantum networks. Our experiments with FeGa nanogratings have also shown the technological feasibility of fabricating vdW layers on ferromagnetic gratings. Being magnetized, such structures may be used for spin manipulations by phonons in the vdW layer without an external magnetic field.^39^

## Supporting Information Available

Nanofabrication of gratings and transfer of vdW flakes, picosecond ultrasonic experiment, theory of phonon mode hybridization, numerical calculations (PDF)
