# Supplementary material for: Coherent Phononics of van der Waals Layers on Nanogratings
Source: Nano Lett. 2022 Aug 12;22(16):6509–15. doi: 10.1021/acs.nanolett.2c01542 (PMC9413225; doi:10.1021/acs.nanolett.2c01542)
Supplement: Supplementary file 1 — nl2c01542_si_001.pdf [file nl2c01542_si_001.pdf]

Coherent phononics of van der Waals layers on nanogratings

Wenjing Yan[1], Andrey V. Akimov[1*], María Barra-Burillo[2], Manfred Bayer[3], Jonathan Bradford[1], Vitalyi E. Gusev[4], Luis E. Hueso[2,5], Anthony J. Kent[1], Serhii M. Kukhtaruk[6], Achim Nadzeyka[7], Amalia Patanè[1], Andrew W. Rushforth[1], Alexey V. Scherbakov[3], Dmytro D. Yaremkevich[3] and Tetiana L. Linnik[6]

[1]*School of Physics and Astronomy, University of Nottingham, Nottingham NG7 2RD, United Kingdom*

[2]*CIC nanoGUNE BRTA, Tolosa Hiribidea, 76, 20018 Donostia-San Sebastián, Basque Country, Spain*

[3]*Experimentelle Physik 2, Technische Universität Dortmund, Otto-Hahn-Str. 4a, 44227 Dortmund, Germany.*

[4]*Laboratoire d'Acoustique de l'Uiversité du Mans (LAUM), UMR 6613, Institut d'Acoustique - Graduate School (IA-GS), CNRS, Le Mans Université, 72085 Le Mans, France*

[5]*IKERBASQUE, Basque Foundation for Science, 48013 Bilbao, Basque Country, Spain*

[6]*Department of Theoretical Physics, V.E. Lashkaryov Institute of Semiconductor Physics, Pr. Nauky 41, 03028 Kyiv, Ukraine.*

[7]*Raith GmbH, Konrad-Adenauer-Allee 8, 44263 Dortmund, Germany*

* e-mail: andrey.akimov@nottingham.ac.uk

Supporting Information

1 Nanofabrication of gratings and transfer of vdW flakes.

The following nanogratings were fabricated on: (1) 100 nm thick FeGa film grown epitaxially on GaAs substrate (ridge/groove: 100/100 nm; 75/75nm,); (2,3) Si substrate with and without thermally evaporated 40-nm thick Cr (ridge/groove: 30/20 nm; 60/40 nm; 120/80 nm; 150/100 nm); (4) 300 nm thick SiO_2 on doped Si substrate (ridge/groove: 75/50nm; 100/60nm; 120/80nm; 150/100nm). Nanogratings (1)-(3) were fabricated by focused ion beam milling (Raith VELION) using Ga^+ beam current of 22 pA [nanograting (1)] and 6 pA [nanogratings (2) and (3)] at 35 kV beam voltage. The milling dose was 0.3 nC μm^{-2} [nanograting (1)], 0.1 nC μm^{-2} [nanograting (2)] and 0.05 nC μm^{-2} [nanograting (3)] and the resulting milling depths were 20-30 nm for all the nanogratings. Structures of nanogratings (4) were defined by electron beam lithography at 20 kV beam voltage. A Cr (5 nm)/Al (40 nm) film were then deposited by electron beam and thermal evaporation, respectively, in ultrahigh vacuum conditions on the defined nanofeatures, followed by a lift-off procedure to create a metal etching mask for SiO_2. SiO_2 was then etched away using reactive ion etching to create the ridge/groove structure using the metal mask. The mask was then removed by immersing the sample in Cr etchant solution.

The bulk (2H) MoS_2 crystal was purchased from HQ graphene. Flakes were obtained by mechanical exfoliation using non-UV dicing tape 6000 series from Loadpoint [S1]. The obtained flakes on the dicing tape were then transferred onto the polydimethylsiloxane tape (PDMS, GElpak PF GEL film WF x 4, 17 mil) to form the MoS2/PDMS stamp. After identifying the flake with desired thickness using optical contrast, it is then transferred onto a specific place on the nanograting using the deterministic transfer technique in reference [2]. The thicknesses of the flakes were then determined by tapping mode atomic force microscopy in air (Asylum Research Cypher S) using Si cantilevers with spring constant of 2 Nm^{-1} (SCOUT 70 RAl, NuNano).

2. Picosecond ultrasonic experiment*:*

For pump-probe experiments with pump excitation from the side of MoS_2 (see Figure 1a) two mode-locked Spectra-Physics Tsunami lasers with wavelength ~ 780 nm, repetition rate ~80 MHz and 120 fs duration were used

to generate pump and probe laser pulses. Picosecond temporal resolution is achieved through the temporal scanning of pump and probe laser with a frequency offset of 800 Hz using the asynchronous optical sampling technique (ASOPS). The polarisation of the pump and probe laser are set to be orthogonal to each other before they get focused onto the sample using the same 50× micro objective, achieving a spot size of around ~1 μm. To avoid the damage to the sample, the laser power was kept < 0.5 mW and < 0.3 mW for pump and probe pulses respectively. The pump-probe signal is then detected by a 10 MHz-bandwidth Newport photodiode.

In the samples with FeGa nanograting, with pump excitation from the back (Figure S1), the pump laser at 1080 nm wavelength is focused onto the sample with 20 × micro objective, giving a spot size of 2.5 μm. The probe laser with 780 nm wavelength is focused onto the surface of the MoS_2 layer with 100 × micro objective resulting in a spot size of 0.6 μm. For both lasers, the given sizes correspond to the full diameter at half maximum of the Gaussian intensity distribution. The typical powers used for pump and probe laser are 100 mW and 0.3 mW respectively. Picosecond temporal resolution and signal detection are also realized by menas of ASOPS with 800 Hz frequency offset and a 10 MHz-bandwidth Newport photodiode, respectively.

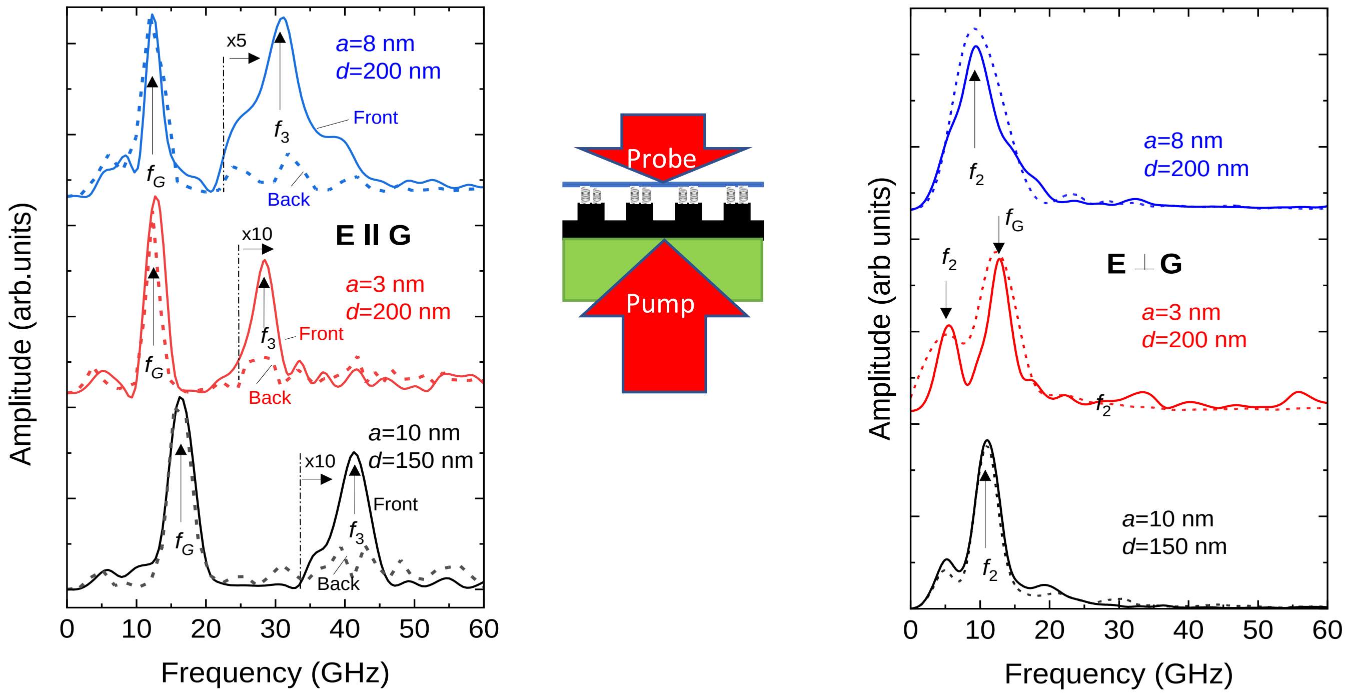

Figure S1. Examples of the measured spectra for pump excitation from the side of vdW layer (Front excitation) and from the side of the substrate (Back excitation as shown in the scheme between the panels) for the layers on FeGa nanogratings. In the back geometry, excitation of the vdW layer is by the coherent phonons injected from the nanograting, whose spectrum possesses a high frequency cut-off. It is seen that there for phonons with $f = f_3$ the spectral peak has essentially lower amplitude in the back geometry (compare solid and dashed curves in **a**) while in **b** it is seen that phonons with $f = f_2$ are excited in the vdW layers in both type of experiments. From these experiments and corresponding analysis (for details see [S3]) we may conclude that the spring stiffness has a value between 10^{17} and 10^{18} N m^{-3}.

The analysis of polarization dependence would require knowledge of the photoelastic tensor and exact distribution of the optical field in the grating. Both tasks require additional experiments and calculations, which are beyond the scope of this work. Polarization models are also hard to find in the literature for the gratings without 2D layers [4]. However, we note that the f_3 mode is dominated by the compression/dilatation elastic strain, while the hybridized modes at frequencies f_1 and f_2 are dominated by the shear elastic strain.

3. Theory of phonon mode hybridization.

Analytical theory of flexural phononic crystal. The hybrid periodic nanostructure studied is a phononic crystal for the laterally propagating acoustic waves. The equations of motion for the vertical displacement component u of the nanolayer in the regions over the grooves and ridges are [S5]:

$$\rho a \frac{\partial^2 u}{\partial t^2} = D \frac{\partial^4 u}{\partial x^2} \text{ , when } -d/4 < x < d/4$$

$$\rho a \frac{\partial^2 u}{\partial t^2} = D \frac{\partial^4 u}{\partial x^2} - \eta u, \text{ when } d/4 < x < 3d/4,$$

where x=0 corresponds to the center of the groove. Here, ρ, a and D denote the density, thickness, and modulus of flexural rigidity, while η stands for the spring rigidity per unit surface area. The solution for monochromatic wave is $u = \tilde{u} \exp{(i\omega t)}$ where the amplitude $\tilde{u}$ of the first equation is

$$\tilde{u} = C_1 \cos(q_g x) + C_2 \cosh(q_g x),$$

where $q_g = \sqrt[4]{\omega^2 \rho a / D}$ is a wavevector for the flexural wave above the groove, $\omega = 2\pi f$. The solution above the ridge depends on the ratio of ω/ω_η, where $\omega_\eta = \sqrt{\eta/\rho a}$ is the circular frequency of the laterally unmodulated mass-on-spring oscillations of the layer connected to the ridge by the springs. For $\omega < \omega_\eta$ the amplitude is

$$\tilde{u} = C_3 \cos[q_t(x - d/2)] \cosh[q_t(x - d/2)] + C_4 \sin[q_t(x - d/2)] \sinh[q_t(x - d/2)]$$

where $q_t = \sqrt[4]{(\omega_\eta^2 - \omega^2)\rho a / D}$. For $\omega > \omega_\eta$

$$\tilde{u} = C_3 \cos[q_t(x - d/2)] + C_4 \cosh[q_t(x - d/2)] \text{ ,}$$

where $q_t = \sqrt[4]{(\omega^2 - \omega_\eta^2)\rho a / D}$.

The four-component displacement fields in the regions above the grooves and ridges of the hybrid nanostructure are coupled by the boundary conditions at the interfaces, which require continuity of the mechanical displacement and of its three first derivatives [S6]. The boundary conditions allow us to write the system of linear equations for the coefficients C_i and finally find the dependence of phonon mode frequency ω on the grating vector $G=2\pi/d$:

For $\omega < \omega_\eta$

$$\begin{bmatrix} \cos\mathbf{1} & \cosh\mathbf{1} & -\cos\mathbf{2}\cosh\mathbf{2} & -\sin\mathbf{2}\sinh\mathbf{2} \\ -q_g\sin\mathbf{1} & q_g\sinh\mathbf{1} & q_t(\cos\mathbf{2}\sinh\mathbf{2} - \sin\mathbf{2}\cosh\mathbf{2}) & q_t(\cos\mathbf{2}\sinh\mathbf{2} + \sin\mathbf{2}\cosh\mathbf{2}) \\ -q_g^2\cos\mathbf{1} & q_g^2\cosh\mathbf{1} & 2q_t^2\sin\mathbf{2}\sinh\mathbf{2} & -2q_t^2\cos\mathbf{2}\cosh\mathbf{2} \\ q_g^3\sin\mathbf{1} & q_g^3\sinh\mathbf{1} & -2q_t^3(\cos\mathbf{2}\sinh\mathbf{2} + \sin\mathbf{2}\cosh\mathbf{2}) & 2q_t^3(\cos\mathbf{2}\sinh\mathbf{2} - \sin\mathbf{2}\cosh\mathbf{2}) \end{bmatrix} \begin{bmatrix} C_1 \\ C_2 \\ C_3 \\ C_4 \end{bmatrix} = \begin{bmatrix} 0 \\ 0 \\ 0 \\ 0 \end{bmatrix},$$

and for $\omega > \omega_\eta$

$$\begin{bmatrix} \cos\mathbf{1} & \cosh\mathbf{1} & -\cos\mathbf{2} & -\cosh\mathbf{2} \\ -q_g\sin\mathbf{1} & q_g\sinh\mathbf{1} & -q_t\sin\mathbf{2} & q_t\sinh\mathbf{2} \\ -q_g^2\cos\mathbf{1} & q_g^2\cosh\mathbf{1} & q_t^2\cos\mathbf{2} & -q_t^2\cosh\mathbf{2} \\ q_g^3\sin\mathbf{1} & q_g^3\sinh\mathbf{1} & q_t^3\sin\mathbf{2} & q_t^3\sinh\mathbf{2} \end{bmatrix} \begin{bmatrix} C_1 \\ C_2 \\ C_3 \\ C_4 \end{bmatrix} = \begin{bmatrix} 0 \\ 0 \\ 0 \\ 0 \end{bmatrix}.$$

There is no solution for $\omega = \omega_\eta$. The compact notations introduced in the above systems of equations are: $q_q d/4 \equiv \mathbf{1}$, and $q_t d/4 \equiv \mathbf{2}$.

The results for normalized frequency $\Omega = \omega/\omega_\eta$ for the three lowest modes are presented in Fig. 4a as a function of normalized grating vector $\bar{G} \equiv \sqrt[4]{D/\eta} G$. More phonon modes are shown in Figure S2. The asymptotics

for small and large G are shown in Figure S3. The calculated dependence of the frequency on the layer thickness $f(a)$ is shown in Figure 4b, where for the galfenol film with thickness 10 nm the value D=7.85×10^{-15} kg m^{2}s^{-2} was used.

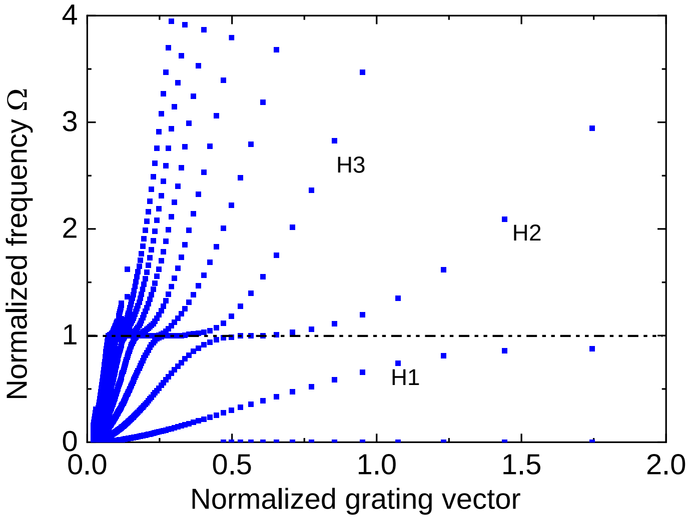

Figure S2. Calculated dependences $\Omega(\bar{G})$ for even low- and high-frequency branches of phonon modes. The modes H1, H2 and H3 graph are the same as in Fig. 4b and more modes are shown in the present figure. All modes show the hybridization near Ω=1, but the avoided crossing gap decreases with the increase of the phonon mode order number.

Low and high frequency asymptotes for phonon modes

Figure S3 shows the dependence of normalized eigen frequencies on the normalized grating vector. These dependencies are the zeros of determinants given in Methods. Using the solutions for displacement in the regions over the grooves and ridges we can find the asymptotics for frequency dependences. In the long period nanostructures $(\bar{G} \to 0)$, the eigen modes presented by continuous black curves coincide with the phonon branches for the independent standing waves in the nanolayer:

$\Omega_{g,n} = (2x_n/\pi)^2\Omega_F = (2x_n/\pi)^2\bar{G}^2$, above the grooves

$\Omega_{b,n} = \sqrt{1 + (2x_n/\pi)^4\Omega_F^2} = \sqrt{1 + (2x_n/\pi)^4\bar{G}^4}$, above the ridges.

Here $x_n = \{2.37;\ 5.49;\ 8.64; \dots\}$ are the nonzero roots of equation for standing waves: $\tan x_n = -\tanh x_n, n = 1,2, \dots$ These branches are confined separately in the subsequent half-periods of the nanostructure and emerging, respectively, from the points $\Omega = 0$ and $\Omega = 1$, which are exhibiting the maxima in the density of states. Several lowest modes of these two branches are presented on the Fig. S3 by dotted green and red curves, respectively. The deviation of the hybrid structure eigen modes (black continuous curves) from the dotted curves with increasing $\bar{G}$ is the manifestation of the interactions between lower and upper branches. As a consequence of the interaction, the nature of the eigen modes, which are starting from $\Omega = 1$, changes from vibration dominated to flexure dominated. The nature of the eigen modes with $n > 1$, which are starting from $\Omega = 0$, at first changes from flexure dominated to vibration dominated. However, when $\bar{G} \gg 1$, the influence of the nanolayer coupling with the ridges becomes negligible at $\Omega > 1$ and the modes are dominated by flexural motion of the free standing nanolayer over the full period of the nanostructure. They are asymptotically approaching the modes with the dispersion

$\Omega = (n\bar{G})^2 (n = 1, 2, \dots)$,

i.e., of the flexural waves in the free-standing nanolayer with the wave vectors equal to nG, all folded to the center of the Brillouin zone of the flexural phononic crystal. For $n = 1, 2$ and 3 the latter modes are presented by blue dashed curves on Fig. S3. The nature of the lowest eigen mode, H1, transforms with increasing $\bar{G}$ from purely flexural motion in the half period of the hybrid nanostructure into purely mass-on-spring motion of the nanolayer over the full period of the nanostructure. When $\bar{G} \gg 1$ the frequency of its laterally quasi-homogeneous motion saturates at about 10% below $\Omega = 1$ (at $\Omega \to 2/\sqrt{5}$). Note that the revealed transformations of all the eigen modes between the limits $\bar{G} \to 0$ and $\bar{G} \to \infty$ (from the independent standing waves in two different parts of the nanostructure, i.e., over the grooves and over the teeth, to the modes of the free-standing nanolayer over the complete nanostructure) are accompanied by the

doubling of the volume occupied by them. From the theoretical point of view, the presented dependences of the frequencies of the zone centre eigen modes on the grating vector $\bar{G}$ result from the interaction of the flexural waves in the free-standing nanolayer and the flexural waves in the nanolayer supported by the springs.

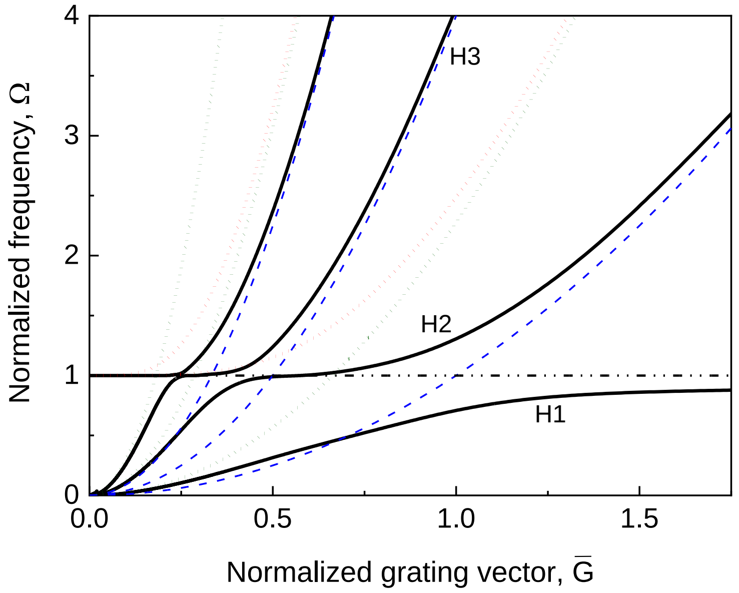

Figure S3. Low and high frequency asymptotes for phonon modes in hybrid nanostructure Solid black lines are analytically calculated normalized eigen frequencies as a function of the normalized grating vector. For low- and high-frequency brunches at low G<<1 these lines coincide with dotted green lines for Ω <1 and dotted red lines for Ω >1. For Ω >1 and $\bar{G} \geq 1$ the mode H1 approaches the value Ω =0.9.

Figure S4 shows the dependence of eigen frequencies on the layer thickness. Here for the normalization of frequency we suggest to use the characteristic frequency $\omega^* = \left(\omega_\eta^2 \omega_F\right)^{1/3}$. Differently to Fig. 4b, where the frequency ω_η was applied for the normalization of the frequency, this characteristic frequency ω^* is favorably independent of the layer thickness. The normalized thickness in Figure S4 is $\bar{a} = \bar{G}^{4/3}$, favorably linearly proportional to the nanolayer thickness. The labels H1, H2 and H3 mark the hybrid eigen modes, which are completely or partially presented in Fig. 4a and 4b in the main text.

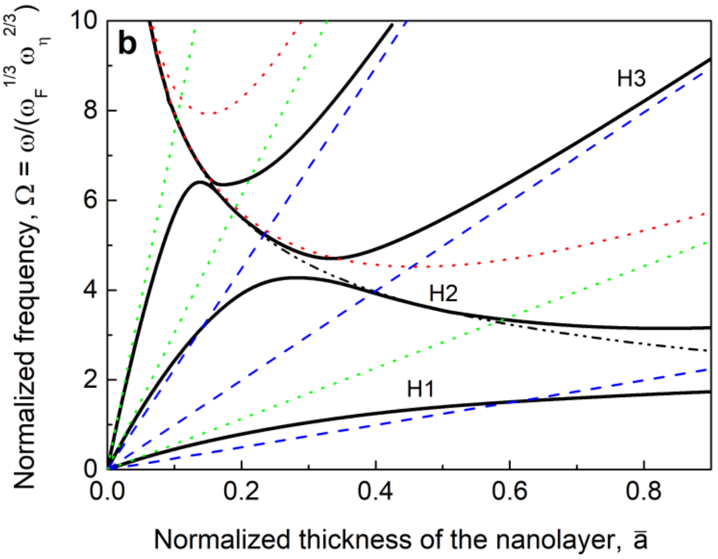

Figure S4. Dependence of eigen frequencies on the layer thickness.

4. Numerical calculations.

We use COMSOL Multiphysics for calculation of the phonon mode spatial distributions and dispersion for MoS_2 possessing an elastic contact with the nanograting ridges. We perform calculations for a unit cell of the flexural phononic crystal and apply Floquet–Bloch periodic boundary conditions: $u_r = u_l e_x^{-iq_x d}$, where u_r and u_l are the atom displacements at the right and left boundaries of the unit cell, respectively, q_x is the Bloch wavenumber. At the mechanically free surfaces, we use stress-free boundary conditions. We introduce thin elastic layers at the interfaces of the grating and flake, that are given by the interaction force $\mathbf{F}_\eta = -\hat{\eta}(\mathbf{u}_\mathrm{u} - \mathbf{u}_\mathrm{b})$, where $\mathbf{u}_\mathrm{u}$ and $\mathbf{u}_\mathrm{b}$ are the atom displacements at the upper and bottom boundaries of the layer, respectively. We consider the diagonal spring stiffness tensor $\hat{\eta}$ with a single nonzero component $\eta_{zz} = \eta$. We use a perfectly matched layer at the backside of the substrate in order to simulate the semi-infinite substrate. The following mass density ρ and stiffness tensor components, C_{ij} for MoS_2 were used: $\rho = 4998.3$ kg m^{-3}, $C_{12} = -54$ GPa, $C_{13} = 23$ GPa, $C_{33} = 52$ GPa and $C_{44} = 18.8$ GPa [S7]. The value of $C_{11} = 200$ GPa, was used to fit the experimental data for S0 mode (see Figure 3a) and differs by ~10% from the values reported in previous works [S8]. The elastic parameters of FeGa and more details about the calculations of phonon spectra in bare FeGa nanogratings may be found in our earlier work [S9].

Figure S5 shows the calculated dispersion of phonon modes in hybrid nanostructures consisting of a van der Waals layer and nanoscale gratings. In accordance with the Bloch-Floquet theorem, the wavenumber of each mode is given by $q_x = \bar{k}_x + (j-1)G$, where $\bar{k}_x$ is a Bloch wavenumber, $G = 2\pi/d$ is a grating wavenumber, and j is a positive integer number. In the weak coupling case (a), the dispersion is similar to the folded dispersion of the free-standing layer. With the increasing spring rigidity η, the dispersion of the lowest modes H1, H2*, and H2 is becoming flat (see (b) and (c)). The dispersion curves of the higher order modes H_j are less affected by the nanogratings, because their frequencies become much larger than mass-on-spring vibration frequency $\omega_\eta = \sqrt{\eta/\rho a}$. As a result, the relative magnitude of the flexural and the mass-on-spring vibration quanta becomes large enough to cause quasi-flexural motion of these modes.

The branches R_j1 and R_j2 correspond to two lowest in frequency surface localized eigenmodes, and their higher harmonics (see e.g. [S9] for details), which are present in our experimental observations, but are not of the main focus of this work. While most modes are well localized in the nanograting and nanolayer, some dispersion curves, e.g., R_21 in (a) and (b), disappear for $\bar{q}d/\pi \gtrsim 0.15$ because corresponding waves become strongly leaking. In the strong coupling case (c), the frequencies of H3 and H3* branches are close to the R_j1 and R_j2 branches causing the hybridization of these modes, which affect both the dispersion and modes profiles. The detailed experimental and theoretical study of these hybrid modes can be done in the future.

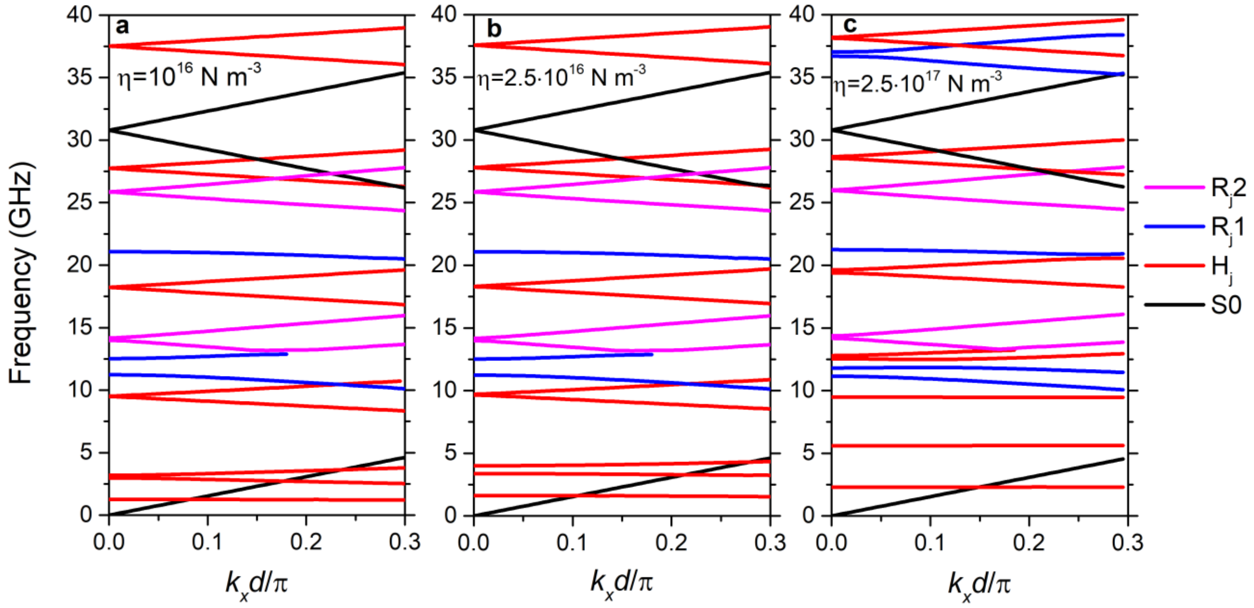

Figure S5. Dispersion of phonon modes in hybrid nanostructures for three different springs rigidities η, corresponding to: (a) week coupling, (b) moderate coupling, and (c) strong coupling between nanolayer and

gratings. Here S_0 and H_j ($j = 1, 2, 3$ ...) were introduced in the main text, and R_j1 and R_j2 correspond to Rayleigh and Sezawa waves, respectively. The nanolayer thickness is fixed to $a = 10$ nm and the grating period to $d = 200$ nm.

Dependence of lowest phonon modes frequencies on lateral rigidity

As it was mentioned in the section Methods, the rigidity is the diagonal tensor

$$\hat{\eta} = \begin{pmatrix} \eta_{xx} & 0 & 0 \\ 0 & \eta_{yy} & 0 \\ 0 & 0 & \eta_{zz} \end{pmatrix}.$$

As far as all phonon modes of interest in this work are sagittal, i.e. with zero y- component of the displacement vector, we can put $\eta_{yy} = 0$ without any loss of generality. Another lateral rigidity component, η_{xx}, is nonzero, in general.

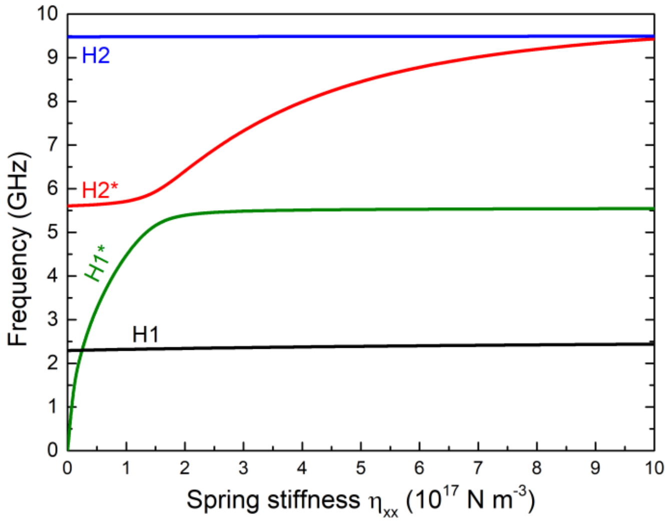

Figure S6. Dependence of lowest phonon modes of the hybrid nanostructures on lateral rigidity η_{xx} at $\eta_{zz} = 2.5 \cdot 10^{17}$ N m^{-3}. The nanolayer thickness is fixed to $a = 10$ nm and the grating period to $d = 200$ nm.

Figure S6 shows the dependence of four lowest phonon modes in the center of the Brillouin zone on the lateral rigidity η_{xx}. One can see that even modes H1 and H2 are weakly dependent on η_{xx}, while the odd modes H1* and H2* are strongly dependent on η_{xx}. Moreover, one can see the avoided crossing between the odd modes. In contrast to the even phonon modes, which motion is mostly parallel to z-direction, the motion of the odd modes is parallel to x-direcion. That is why the odd modes depend on η_{xx}. However, these modes are not excited in our experiments (see e.g. [S9, S10] for details). Therefore, in order to describe our experiments, it is enough to consider only one nonzero component of the rigidity tensor, i.e. η_{zz}. It is worthy of mention that H1* mode is absent in the main text because its frequency tends to zero at $\eta_{xx} \to 0$.

References

S1. Yan, W. et al. Nondestructive Picosecond Ultrasonic Probing of Intralayer and van der Waals Interlayer Bonding in alpha- and beta-In_2Se_3. *Adv. Funct. Mater.* **31**, 2106206 (2021).

S2. Castellanos-Gomez, A. et al. Deterministic transfer of two-dimensional materials by all-dry viscoelastic stamping. *2D Mater.* **1**, 011002 (2014).

S3. Greener, J. D. G. et al. Coherent acoustic phonons in van der Waals nanolayers and heterostructures. *Phys. Rev. B* **98**, 075408 (2018).

S4. Colletta, M.et al. Picosecond ultrasonic study of surface acoustic waves on periodically patterned layered nanostructures. *Ultrasonics* **87**, 126–132 (2018).

S5. Graff, K. F. *Wave Motion in Elastic Solids* (Dover Publ. Inc., New York, 1995).

S6. Doyle, P. F. & Pavlovich, M. N. Vibration of beams on partial elastic foundations, *Earthquake Engineering and Structural Dynamics* **10**, 663474 (1982).

S7. Peelaers, H. & Van de Walle, C. G. Elastic Constants and Pressure-Induced Effects in MoS2. *J. Phys. Chem. C*, **118**, 12073–12076 (2014).

S8. Salasyuk, A. S. et al. Generation of a localized microwave magnetic field by coherent phonons in a ferromagnetic nanograting. *Phys. Rev. B* **97**, 060404 (2018).

S9. Yaremkevich, D. D. et al. Protected long-distance guiding of hypersound underneath a nanocorrugated surface, *ACS Nano*, **15**, 4802–4810 (2021).

S10. Godejohann, F. et al. Magnon polaron formed by selectively coupled coherent magnon and phonon modes of a surface patterned ferromagnet. *Phys. Rev. B* **102**, 144438 (2020).
